# 

**Published:** 1894-04

**Authors:** 


### CONTENTS FOR APRIL, 1894.

ORIGINAL COMMUNICATIONS. PAGE.

Neurasthenia, From the Standpoint of the General Practitioner. By I. N. LOVE, M. D. 513
Artificial Immunity. By HENRY REED HOPKINS, M. D. 527

CLINICAL REPORTS.

Clinical Memoranda from the Surgical Clinic at the Sisters' of Charity Hospital. By HERMAN MYNTER, M. D. 532
A Double Lesion of the Brain—Cerebral Cyst—Cerebellar Tumor. By EDWARD H. ANGELL, M. D. 538

SELECTIONS.

Suppurating Myoma of the Uterine Wall Followed by Twin Pregnancy 542
Rest in Cardiac Affections 543

CORRESPONDENCE.

Frank W. Abbott, M. D. 545

EDITORIAL.

An Object Lesson in Hygiene 546
Topics of the Month 549

Personal 554
Obituary 555
Society Meetings 556
Book Reviews 557
Literary Notes 573

